# Immunomodulators secreted from parasitic helminths act on pattern recognition receptors

**DOI:** 10.3389/fpara.2022.1091596

**Published:** 2023-01-20

**Authors:** Daigo Tsubokawa

**Affiliations:** Department of Parasitology and Tropical Medicine, Kitasato University School of Medicine, Sagamihara, Japan

**Keywords:** helminths, excretory-secretory products, immunomodulators, pattern recognition receptors, therapeutics

## Abstract

Excretory-secretory (ES) products from parasitic helminths contain immunomodulatory molecules, which can regulate host immune responses. These immunomodulatory molecules are crucial for successful parasitism, and play roles in tissue migration, maturation, and reproduction. Some target pattern recognition receptors (PRRs), including toll-like receptor, C-type lectin receptor, receptor for advanced glycation end products, and nucleotide-binding oligomerization domain-like receptor. PRRs trigger activation of signaling cascades, inducing innate inflammatory responses and adaptive immunity in hosts. This article reviews ES immunomodulators identified in parasitic helminths that act on PRRs, and their PRR-facilitated immune-regulatory mechanisms. In addition, we describe the therapeutic potential of ES immunomodulators for allergic and inflammatory diseases.

## Introduction

Parasitic helminths have successfully evolved to reside in almost all vertebrates (Dobson et al., 2008). Nearly one third of the human population, especially children, are affected by parasitic helminths (Hotez et al., 2008). Parasitic helminth infection induces host type 2 immune responses mediated by immune cells (i.e., eosinophils, basophils, type 2 innate lymphoid cells, and T helper type 2 (Th2) cells) and cytokines (i.e., interleukin (IL)-4, IL-5, IL-13), resulting in B cell isotype switching to IgE (Harris and Loke, 2017). Type 2 immune responses have crucial roles in both clearing helminths and host tissue repair (Lloyd and Snelgrove, 2018). Parasitic helminths release excretory/secretory (ES) products containing immunomodulators into hosts. Nematodes secrete ES products from their gastrodermis, which are released from their oral and anal openings. Nematode can also release ES products as body fluid from ES pores on their cuticles. In platyhelminths, ES products are mainly released from their tegument directly. However, it is difficult to distinguish between ES products released from different sites (Drurey et al., 2020). ES products are composed of proteins, glycans, lipids, nucleic acids, small metabolites, and extracellular vehicles (EVs) (Claycomb et al., 2017; Hokke and van Diepen, 2017; Maizels et al., 2018; Wangchuk et al., 2019; Drurey and Maizels, 2021; Yeshi et al., 2022). Innate and adaptive immune cells are manipulated by ES immunomodulators, which affect the balance between type 1 and type 2 immune responses (Maizels and McSorley, 2016; Maizels et al., 2018). Although many ES immunomodulators can induce Th2 cell proliferation and differentiation, inducing type 2 immunity, they can also induce alternatively activated macrophages and regulatory T (Treg) cells, which produce immunosuppressive cytokines (i.e., IL-10 and transforming growth factor-β (TGF-β)). Furthermore, some ES immunomodulators can also directly induce these immunosuppressive cells (Maizels et al., 2018). Parasitic helminths could use ES immunomodulators to overcome and dampen host protective immune responses for successful parasitism. As purification and analysis of natural ES products from parasitic helminths is time-consuming and labor-intensive, many ES products has been recombinantly expressed or chemically synthesized to perform functional studies.

Pattern recognition receptors (PRRs) are major sensors against danger associated molecular patterns (DAMPs) released by damaged host cells and pathogen-associated molecular patterns (PAMPs) derived from various pathogens, including viruses, bacteria, fungi, and parasites (Janeway and Medzhitov, 2002; Rubartelli and Lotze, 2007; Takeuchi and Akira, 2010; Minnicozzi et al., 2011). Immune cells, particularly dendric cells (DCs) and macrophages, express surface and intracellular PRRs including Toll-like receptors (TLRs), C-type lectin receptors (CLRs), the receptor for advanced glycation end products (RAGE), and nucleotide-binding oligomerization domain-like receptors (NLRs) (Lee and Kim, 2007; Sparvero et al., 2009). Recognition of DAMPs and PAMPs by PRRs activates complex intracellular signaling pathways leading to cytokine production, DC maturation, and T cell priming (Iwasaki and Medzhitov, 2004; De Nardo, 2015). Thus, both innate and adaptive immune responses are triggered by PRR-mediated signaling.

PRRs recognizing DAMPs and PAMPs also trigger host immune responses during parasitic helminth infection (Maizels et al., 2018). Parasitic helminths need to manipulate PRR signaling to suppress these protective immune responses and achieve successful migration, maturation, egg production, and longitudinal parasitism. ES immunomodulators which regulate signaling in macrophages and DCs *via* TLRs, CLRs, and NLRs have been identified (Zakeri et al., 2018). Since genes encoding DAMP-mimicking proteins, such as S100-like EF-hand proteins, were predicted from parasitic helminth genomes registered in WormBase Compara (http://parasite.wormbase.org), we infer that they also have effects against PRR signaling. In fact, we have identified a S100-like ES immunomodulator against RAGE (Tsubokawa et al., 2017; Tsubokawa et al., 2019). In this review, we introduce ES immunomodulators against PRRs, explain their mechanisms of host immune response regulation, and discuss their therapeutic potential against immune-related diseases.

## Immune regulation *via* PRRs by ES immunomodulators

ES products from parasitic helminths contain immunomodulators (Maizels et al., 2018; Zakeri et al., 2018) that block PRRs and interfere with macrophage and DC activation (Maizels et al., 2018). PRR expression and downstream intracellular signaling are also altered (Zakeri et al., 2016). Furthermore, alterations in the activation patterns of transcription factors within macrophages and DCs lead to the induction of Treg and Th2 cell differentiation, and inhibition of Th1 cells (Zakeri et al., 2018). Some ES immunomodulators targeting PRRs have been identified, and their immunoregulatory effects have been characterized (**Table 1**). For example, ES-62 and Fh12 from the rodent filarial nematode *Acanthocheilonema viteae*, which parasitize subcutaneous tissues, and the liver fluke *Fasciola hepatica*, respectively, act on TLR4. Both immunomodulators can suppress TLR4-mediated inflammatory responses and induce immune tolerance. Venestatin and SjE16.7 from the rodent intestinal nematode *Strongyloides venezuelensis* and blood fluke *Schistosoma japonicum*, respectively, act on RAGE, but have contrasting effects on RAGE signaling. Most PRR-targeting ES immunomodulators can beneficially stabilize the parasitism of parasitic helminths through suppression and/or alteration of macrophage and DC activity.

**Table 1:** PRR-targeting ES immunomodulators from parasitic helminths.

PRRs targeted	Helminths	ES products	Action and effects in host cells	References
TLR				
TLR4	*A. viteae*	ES-62	Activation of ERK1/2 signaling, degradation of TLR4	(Harnett and Harnett, 2010) (Melendez et al., 2007)
	*F. hepatica*	Fh12	Suppressing TLR4 cofactor CD14 expression	(Martin et al., 2015)
TLR3	*F. hepatica*	Cathepsin L1	Degradation of TLR3	(Donnelly et al., 2010)
TLR2/4	*B. schroederi*	Cysteine protease inhibitors	Polarization of alternative macrophages	(Xu et al., 2022)
CLR				
DC-SIGN/MR	*S. mansoni*	Omega-1	Degradation of host RNAs	(Everts et al., 2012)
RAGE				
	*S. venezuelensis*	Venestatin	Blocking of RAGE signaling, suppressing pro-inflammatory molecules	(Tsubokawa et al., 2021)
	*S. japonicum*	SjE16.7	Inducing production of pro-inflammatory cytokines	(Wu et al., 2020)
NLR				
	*T. muris*	Exosome	Inducing production of Th1 cytokines	(Alhallaf et al., 2018)

### Immunomodulators against TLRs

TLRs are transmembrane receptors, of which 13 subtypes (TLR1–TLR13) have been identified. TLR1-10 and TLR1–TLR13 are present in humans and mice, respectively (Akira, 2003). TLR1, TLR2, TLR4, TLR5, and TLR6 are expressed on the plasma membrane, while TLR3, TLR7, TLR8, and TLR9 are present in endosomal compartments. Myeloid differentiation factor 88 (MyD88) is a common TLR adaptor molecule, other than for TLR3 (O’Neill and Bowie, 2007). Toll/interleukin-1 receptor-domain-containing adapter-inducing interferon-β (TRIF) is included in the MyD88 independent pathway of TLR3 and TLR4 signaling (Yamamoto et al., 2003). MyD88 and TRIF activation induces the production of pro-inflammatory cytokines IL-12, IL-6, and tumor necrosis factor (TNF)-α (De Nardo, 2015). The mitogen-activated protein kinase (MAPKs) pathway, including extracellular signal-related kinases 1 and 2 (ERK1/2), c-jun NH2-terminal kinase (JNK), and p38MAPK, is the main downstream signaling cascade of MyD88 and TRIF. They activate transcription factors including nuclear factor-κB (NF-κB), activating protein 1 (AP-1), and interferon regulatory factors, thereby inducing cytokine production and DC and macrophage activation (Arthur and Ley, 2013). Type 2 immune responses and DC hypo-responsiveness are mainly induced by ERK1/2 signaling, while type 1 responses are induced by JNK and p38 signaling (Agrawal et al., 2003; Dunand-Sauthier et al., 2011). ES immunomodulators mainly target TLR2 and TLR4 to activate ERK1/2 signaling. However, a comprehensive picture of the mechanisms underlying type 2 immunity induction and/or immunotolerance remains to be elucidated.

ES-62 is a multifunctional glycoprotein secreted by *A. viteae* and may function as a peptidase (Harnett et al., 2012; Harnett, 2014). ES-62 induces TLR4 degradation (Melendez et al., 2007) and MyD88 sequestration to inhibit TLR signaling (Pineda et al., 2014; Ball et al., 2018). Further, ES-62 activates ERK1/2 signaling *via* TLR4 on DCs and macrophages (Harnett and Harnett, 2010). Consequently, ES-62 suppresses Th1-inducing IL-12 production and DC activation, and induces type 2 immune responses and IL-10-dependent anti-inflammatory responses. Phosphorylcholine (PC), with N-linked glycans, on ES-62 is responsible for its interactions with TLR4 and immunomodulatory activities (Goodridge et al., 2007; Harnett et al., 2012).

*Baylisascaris schroederi*, a roundworm of giant pandas, secretes cysteine protease inhibitors. The recombinant cysteine protease inhibitor induces macrophage polarization to alternatively activated subtypes through TLR2/4, leading to type 2 immunity and immunosuppression (Xu et al., 2022).

*F. hepatica* secretes Fh12, a fatty acid binding protein. Fh12 can bind to the TLR4 cofactor CD14 and suppress its expression and subsequently, TLR4 signaling (Martin et al., 2015). Recombinant Fh12 acts on various TLRs and suppresses macrophage activation (Ramos-Benítez et al., 2017), and also induces alternatively activated macrophages. *F. hepatica* secretes cathepsin L1, a cysteine protease. Recombinant cathepsin L1 inhibits TRIF signaling by degrading TLR3 (Donnelly et al., 2010). A recombinant cysteine protease protein from *Clonorchis sinensis*, another liver fluke, downregulates IL-12 expression though the TLRs/Myd88/MAPKs/NF-κB pathways (Xie et al., 2022).

SJMHE1, a synthetic peptide derived from heat shock protein 60 of *S. japonicum*, primes Tregs *via* TLR2-dependent ERK1/2 signaling on DCs and macrophages (Wang et al., 2009). The phospholipid lysophosphatidylserine (lyso-PS) expressed in the tegument of *S. mansoni* also activates DCs *via* TLR2 to promote Treg differentiation (van der Kleij et al., 2002; Retra et al., 2015). Lacto-N-fucopentaose III (LNFPIII), found in soluble *S. mansoni* egg antigens, induces ERK1/2 signaling *via* TLR4, leading to type 2 immune responses like ES-62 (Thomas et al., 2003; Thomas et al., 2005). Further studies are needed to determine whether these immunomodulators are constitutively present in the ES products of *Schistosoma.*

### Immunomodulators against CLRs

CLRs (i.e., DC-specific ICAM-3 grabbing nonintegrin (DC-SIGN), mannose receptor (MR), and Dectin-1) are expressed on the cell surface, recognize a variety of glycans, and can act as endocytic and/or signaling receptors (Brown et al., 2018). CLR signaling can also affect the TLR signaling pathway, leading to altered inflammatory responses (Trinchieri and Sher, 2007). Since parasitic helminths produce glycoconjugates, such as glycoproteins and glycolipids (Hokke and van Diepen, 2017), interactions between helminth-derived glycans and CLRs on host immune cells are important in regulating the innate and adaptive immune responses (Tundup et al., 2012). However, glycoconjugates from ES products that act as CLR ligands require further characterization.

ES products from *F. hepatica* induce the production of immunosuppressive cytokines IL-10 and TGF-β *via* MR and Dectin-1 (Guasconi et al., 2011; Guasconi et al., 2018), although the specific immunomodulators against CLRs remain to be identified. *F. hepatica* also secretes serine protease inhibitors (serpins). Recombinant serpins regulate mannose binding lectin-associated serine proteases, thereby preventing the lectin complement pathway (De Marco Verissimo et al., 2022). However, it is unknown whether serpins perform regulatory functions against CLR signaling.

In the case of *Schistosoma*, lyso-PS is engaged by Dectin-1 on DCs, which induces IL-10 production and Treg priming (Dillon et al., 2006). LNFPIII suppresses DC activation *via* MR and DC-SIGN (Harnett and Harnett, 2010; Bhargava et al., 2012). Omega-1, a glycoprotein secreted from *S. mansoni* eggs, has T2 ribonuclease (RNase) activity and is recognized by DC-SIGN and MR on DCs. Both natural and recombinant omega-1 can induce type 2 immune responses (Everts et al., 2009; Wilbers et al., 2017). The MR can capture and internalize omega-1, leading to Th2 polarization by degrading host RNA (Everts et al., 2012).

ES immunomodulators against CLRs from parasitic nematodes currently remain to be identified. ES proteins from the rodent whipworm *Trichuris muris* bind to MR on macrophages, and may induce macrophage activation *via* IL-6 production (deSchoolmeester et al., 2009). Glycoconjugates from the roundworm *Ascaris suum* extracts are recognized by DC-SIGN and MR, and can suppress DC maturation and inflammatory responses (Favoretto et al., 2017).

### Immunomodulators against RAGE

RAGE is highly expressed on the cell surfaces of fibroblasts and endothelial cells in the skin, alveolar epithelial cells in lungs, and various immune cells (Teissier and Boulanger, 2019; Guarneri et al., 2021). RAGE mainly recognizes AGEs and DMAPs, such as S100 small calcium-binding proteins and high-mobility group box 1 protein. RAGE signaling induces MAPK phosphorylation, followed by NF-κB, AP-1, and Stat3 activation (Byun et al., 2017; Kim et al., 2021). Subsequently, pro-inflammatory cytokines and endothelial adhesion molecules are expressed, which trigger inflammatory responses (Schmidt et al., 2001). Although RAGE-mediated inflammatory responses relate to the progression of various diseases, such as Alzheimer’s disease, rheumatoid arthritis, asthma, ulcerative colitis, and diabetes (Sims et al., 2010), little is known about the role of RAGE against parasitic helminth infection.

We identified a S100-like EF-hand Ca^2+^-binding protein, venestatin, that contains two EF-hand Ca^2+^ binding-domains, from the ES products of *S. venezuelensis* (Tsubokawa et al., 2017). Recombinant venestatin binds to RAGE, but not TLR4, in a Ca^2+^-dependent manner (Tsubokawa et al., 2019). Experiments using venestatin-knockdown *S. venezuelensis* larvae indicated that endogenous venestatin suppresses RAGE-mediated inflammatory responses, including TNF-α, cyclooxygenase-2, intercellular adhesion molecule-1, and vascular cell adhesion molecule-1 expression (Tsubokawa et al., 2021). Further, macrophage and neutrophil infiltration was also suppressed by endogenous venestatin in the skin of hosts after larval infection. Venestatin is conserved across several nematode species, implying that the suppression of RAGE-mediated inflammation by venestatin might be a general mechanism underlying parasitic nematode infection. Another EF-hand Ca^2+^-binding protein, SjE16.7, is secreted from *S. japonicum* eggs. Recombinant SjE16.7 binds with RAGE and promotes inflammatory responses by producing the pro-inflammatory cytokines TNF-α and IL-6 (Wu et al., 2020). Comparative structural studies between RAGE-binding proteins from parasitic helminths will help elucidate the functional differences between venestatin and SjE16.7, and their roles in helminth infection.

### Immunomodulators against NLRs

The NLR family, which include the NOD, NLRP, and IPAF subfamilies, constitute cytosolic sensors and inflammasomes. NLRP3 is a most characterized component of inflammasomes. DMAPs and/or PMAPs trigger NLRP3 inflammasome complex formation, which contain procaspase-1 (Schroder and Tschopp, 2010). Caspase-1 autoactivation *via* procaspase-1 clustering induces the release of IL-18 and IL-1β, triggering inflammatory responses in immune cells (Awad et al., 2018).

Although there is limited knowledge on the effects of ES products on NLRs, a synthetic analog of ES-62 is known to downregulate inflammasome-mediated signaling in macrophages (Rzepecka et al., 2015). However, it is unclear whether this analog can be internalized in host cells and directly affect inflammasome activation. Recently, it has been revealed that EVs secreted from *T. muris* can suppress type 2 immune responses by inducing the production of Th1-inducing IL-18 *via* NLRP3 (Alhallaf et al., 2018). Components of EVs from parasitic helminths may be attractive candidates for NLR ligands since EVs can be incorporated into host cells and act as cargo for intercellular communication (Drurey et al., 2020).

## Therapeutic potential of ES immunomodulators *via* PRRs

**Figure 1:**
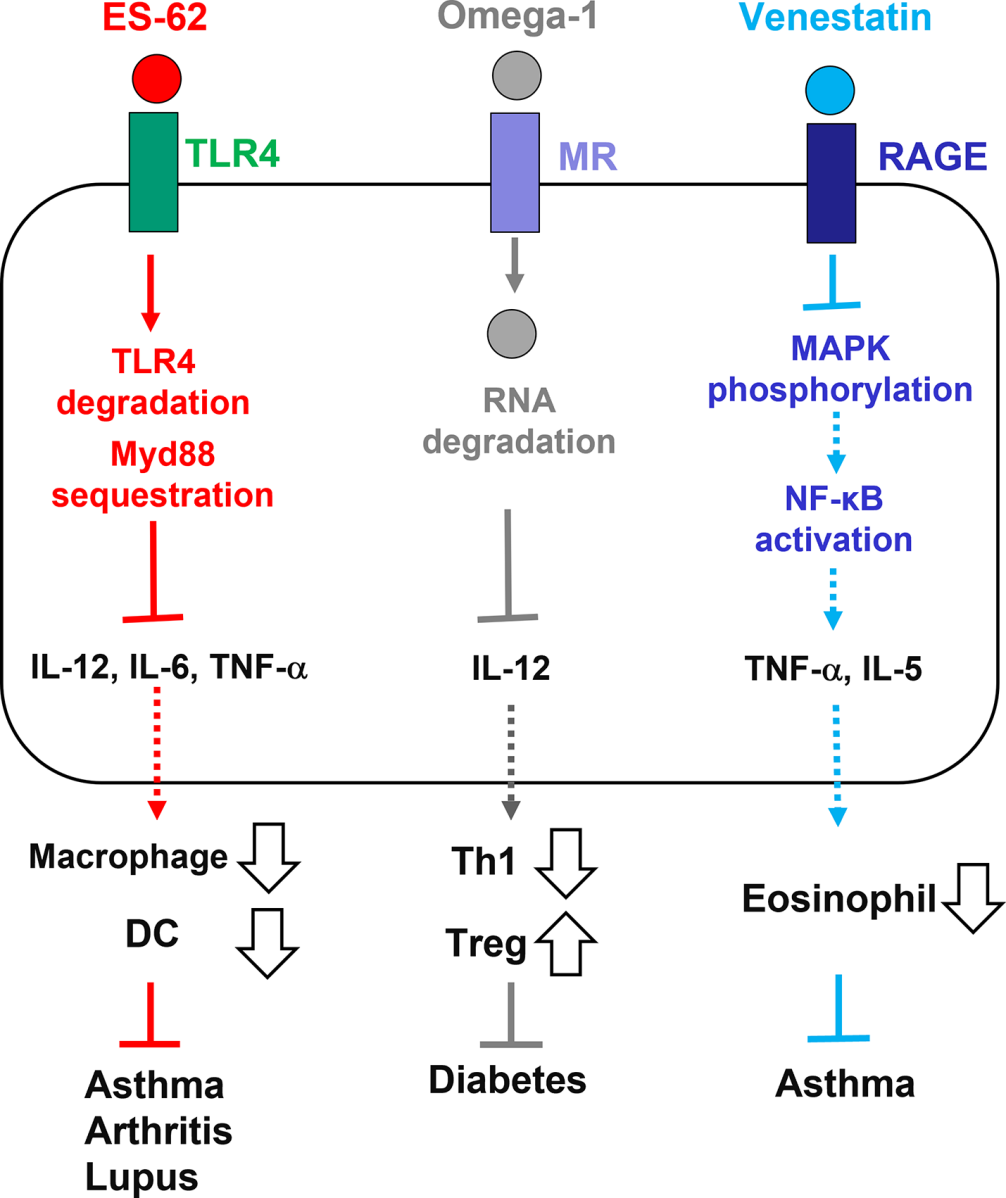
Therapeutic potential of ES immunomodulators from parasitic helminths which act on PRRs. ES-62 from *Acanthocheilonema viteae* acts on TLR4, inducing its degradation and MyD88 sequestration. Pro-inflammatory cytokine production and DC/macrophage activation are suppressed by ES-62 action. Omega-1 from *Schistosoma mansoni* is captured and internalized by MR, inducing host RNA degradation and suppressing IL-12 production. Tregs, but not Th1 cells, are induced by omega-1 action. Venestatin from *Strongyloides venezuelensis* blocks RAGE signaling, suppressing MAPK phosphorylation and NF-κB activation. TNF-α and IL-5 production and eosinophilia are suppressed by venestatin action. These actions may alleviate inflammatory diseases in mouse models. PRR, pattern recognition receptor; ES, excretory-secretory; TLR, toll-like receptor; MR, mannose receptor; RAGE, receptor for advanced glycation end-products; IL, interleukin; TNF, tumor necrosis factor; DC, dendric cell; Treg, regulatory T cell; Th1, T helper type 1 cell; MAPK, mitogen-activated protein kinase; MyD88, myeloid differentiation factor 88; NF-κB, nuclear factor-κB.

ES products from parasitic helminths have therapeutic effects against allergic and autoimmune diseases (Smallwood et al., 2017; Maizels, 2020). The therapeutic potential of a few ES immunomodulators that act *via* PRRs have been elucidated to date (**Figure 1**). *A. viteae* ES-62 and its PC-based small molecule analogs interfere with the progression of pathological processes in mouse models of asthma, rheumatoid arthritis, and systemic lupus erythematosus (Rzepecka et al., 2014; Aprahamian et al., 2015; Doonan et al., 2018). TLR4 degradation and MyD88 sequestration by ES-62 suppresses inflammatory responses, such as DC and macrophage activation and the production of pro-inflammatory cytokines, which affects pathological processes. *S. mansoni* omega-1 protects against type 1 diabetes in mice by internalization *via* MR and degradation of RNAs in DCs, followed by prevention of Th1-mediated IL-12 production and induction of Treg priming (Zaccone et al., 2011; Everts et al., 2012). The *C. sinensis* cysteine protease ameliorates colonic inflammation in dextran sulfate sodium-induced colitis mouse models by downregulating IL-12 expression *via* TLR signaling (Xie et al., 2022). *S. venezuelensis* venestatin and EF-hand domain-based small peptides can prevent pathogenesis in house dust mite-induced allergic asthma mouse models by interfering with the RAGE/MAPKs/NF-κB pathway and suppression of TNF-α and IL-5 production (Tsubokawa and Satoh, 2022). Since PRRs are attractive therapeutic targets for inflammatory diseases (Paul-Clark et al., 2012; Sukkar et al., 2012), further studies should be performed to develop clinical applications of these ES immunomodulators.

## Conclusion

ES products secreted from parasitic helminths contain various immunomodulators against PRRs. The ES immunomodulators may have potential therapeutic benefits in PRR signaling-related inflammatory diseases such as allergic and autoimmune diseases (Minnicozzi et al., 2011). In past decades, the functional analysis of recombinantly expressed ES proteins have helped to identify the possible therapeutic effects of ES immunomodulators. However, a limited number of ES immunomodulators have been identified and characterized to date due to the laborious purification and production processes needed to obtain individual molecules from ES products. Recent advances in high-throughput screening methods to identify anti-inflammatory molecules from ES products will accelerate the discovery of drug candidates against PRRs from parasitic helminths (Ryan et al., 2022).

## Author contributions

The author confirms being the sole contributor of this work and has approved it for publication.
